# Effect of hydroalcoholic leaves extract of *Indigofera spicata* Forssk. on blood glucose level of normal, glucose loaded and diabetic rodents

**DOI:** 10.1186/s12906-015-0852-8

**Published:** 2015-09-11

**Authors:** Eshetie Melese Birru, Mohammedbrhan Abdelwuhab, Zewdneh Shewamene

**Affiliations:** Department of Pharmacology, College of medicine and health sciences, University of Gondar, Gondar, Ethiopia

**Keywords:** Diabetes mellitus, Alloxan, *Indigofera spicata*, Blood glucose level

## Abstract

**Background:**

Diabetes mellitus is found in all parts of the world and is rapidly increasing in its coverage with alarming rate especially in Asia and Africa. Research is increasingly done with the aim of developing a relatively safe and efficacious anti-diabetic plant based products. Parallelly, this investigation was carried out to evaluate the effect of the hydro alcoholic leaves crude extract of *Indigofera spicata (ISP)* on the blood glucose level(BGL) of normoglycemic, oral glucose loaded and alloxan induced diabetic rodents.

**Methods:**

The animals were randomly divided into five groups (*n* = 6) for all the aforementioned three models. In all models, group-I mice provided 2%tween-80, group-II were treated with 5 mg/kg glibenclamide and the remaining three groups (III, IV & V) were treated with 100, 200, and 400 mg/kg dose of the extract respectively. Statistical significance of differences in BGLs within and between groups was analyzed by SPSS version-21 using one way ANOVA followed by Tukey’s post hoc multiple comparison.

**Result:**

200 mg/kg and 400 mg/kg extract treated groups of normoglycemic mice showed significant (*p* < 0.05) BGL reduction compared to the pre-exposure level. In case of OGTT model BGL reduction was statistically significant (*p* < 0.05) in only 400 mg/kg exposed groups at the 120 min of post-exposure compared to the initial level. However, the BGL reducing effect of doses of the extract at the 4^th^, 6^th^ and 10^th^ hours of post treatment on diabetic mice was found statistically significant compared to both the negative control (*p* < 0.001) and their respective pretreatment levels (*p* < 0.05).

**Conclusion:**

As it is claimed in ethnobotanical studies, the hydroalcoholic crude extract of *ISP* leaves have shown prominent anti-diabetic effect and can be therefore used as a good insight for new anti-diabetic drug source with a call for further studies.

## Background

Diabetes mellitus could be defined as a metabolic disorder of multiple etiology characterized by chronic hyperglycemia with disturbances of carbohydrate, fat and protein metabolism resulting from defects in insulin secretion, insulin action, or both [1]. The disease is found in all parts of the world and is rapidly increasing in its coverage [2, 3]. The total number of people with diabetes is estimated to rise from 285 million in 2010 to 439 million or more, that is predicted to be >7.7 % fro*m the world’s adult* population in 2030. Regions with greatest potential are Asia and Africa, where diabetes mellitus rates could rise to two to three-folds than the present rate [4, 5].

Although efforts to control hyperglycemia and associated clinical symptoms are important, the main confronts in successfully managing the patient with diabetes mellitus are targeted at reducing or preventing complications, and improving life expectancy and quality of life [6]. Moreover, currently the available therapies only partially compensate for metabolic abnormalities seen in diabetics and don’t optimally correct the fundamental biochemical changes and even not efficient to correct the course of diabetic complications [7]. Clinically, there is also significant treatment failures, untoward side effects and enormous cost associated with oral anti-diabetic drugs generating an urgent need and desire for alternative treatments [8].

Despite the introduction of many anti-hyperglycemic agents from natural and synthetic sources, diabetes and its secondary complications continue to be a major clinical challenges. Not only in the past several decades but also currently the search for more effective and safe antihyperglycemic agents has continued to be an area of research interest to expand the therapeutic armamentarium [9, 10]. As the number of people with diabetes multiplies nationally and worldwide, the disease takes an ever-increasing proportion of national and international health care budgets.

Currently like streptozotocin, alloxan-induced diabetes is one of the widely used model to induce Type I diabetes mellitus and study hypoglycemic activity in animal models. Though, alloxan has multiphasic effect on the blood glucose level in its early course of action, permanent diabetic hyperglycemia could be induced within 24–48 h after administration. And this is due to the selective pancreatic beta cell toxicity of alloxan. Surprisingly, the non-beta cells and other endocrine and non-endocrine islet cell types along with extrapancreatic parenchyma remain intact, providing the evidence of selective toxic action of alloxan [11–13].

Species of the genus *Indigofera* like *Indigofera pulchra* [14], *Indigofera tinctoria* [15], *Indigofera arrecta* [16] and *Indigofera stachyodes* [17] have experimentally demonstrated hypoglycemic/antidiabetic effect with greater margin of safety. It is stated that diabetes is oxidative stress disorder [18] and hyperglycemia is known in mediating oxidative damage and impairing the endogenous antioxidant defense systems in many ways during diabetes in addition to generating free radicals. Thus, this led to that drugs that can improve glycemic index and/or oxidative stress will be beneficial in the treatment of diabetes mellitus and its complications [19, 20]. In addition substances with cytotoxic activities are also therapeutically recommended for their antidiabetic effect [21, 22]. In line to these, the bioactive constituents of *Indigofera spicata* has verified cytotoxic and antioxidant activities *in vitro* [23]. Furthermore, *Indigofera spicata* Forssk. have been used in treatment of diabetes mellitus and chronic illness as claimed in literature [24, 25] and people use the plant material locally. But unlike the previous species of genus *Indigofera* systemic pharmacological studies have not been yet reported to support this claim. Accordingly, this study has been taken up to investigate the effect of the crude hydroalcoholic leaves extract of *indigofera spicata* Forssk*.* on blood glucose level of normal, oral glucose loaded and alloxan-induced diabetic rodents.

## Methods

### Plant material collection

Fresh leaves of the plant were collected from Zegie peninsula (southern part of Lake Tana, Ethiopia) where people commonly use the plant for treatment of different health problems at 10^th^ October, 2012. Taxonomic identification and authentication was done at the National Herbarium, Department of Biology, Science Faculty, Addis Ababa University and a voucher specimen is already deposited with EM001.

### Chemicals and instruments

Alloxan(Sigma Aldrich, Germany), Glibeneclamide(Cadila pharmaceuticals, Ethiopia), normal saline(Epharm, Ethiopia), Tween-80(Avishkar Lab Tech chemicals, India), methanol(Avishkar Lab Tech chemicals, India), ethanol(supertek chemicals, India), hydrochloric acid(supertek chemicals, India), chloroform(Avishkar Lab Tech chemicals, India), sulfuric acid(Supertake, India), Nitric acid(supertek chemicals, India), acetic anhydride(central drug house, India), ferric sulfate(BDH Ltd, England)), ferric chloride(Fisher Scientific Company, New Jersey)), lead acetate(BDH Ltd, England), benzene(Nice laboratory reagent, India), Mayer’s reagent(Avishkar Lab Tech chemicals, India), Wagner’s Reagent(BDH, England), glucose standard strip/kits-ACCU-CHEK® ACTIVE(Roche Diagnostics GmbH, Germany) electronic glucometer, analytical balance, macerating chamber, oven, sieve, gavages, cages.

### Animals used

Healthy Male Swiss albino mice (weighing 20–28 g and age of 6–10 weeks) and wistard rats (weighing 180–230 g and age of 2–3 months) were purchased from Ethiopian public health Institution (EPHI) which was formerly called Ethiopian Health and Nutritional Research Institute, Addis Ababa. Female rodents were excluded for greater compatibility nature of males for the models and adherence to ethical issues [13, 26] except for toxicity studies. After randomization in to various groups and before initiation of experiment, the mice were acclimatized to the laboratory conditions [27] of the Department of Pharmacology, College of Medicine & Health Sciences, University of Gondar. The selected animals were housed in Polypropylene cages within recommended environmental conditions, feed with fabricated rodent diet and water *ad libtum*. The animals were exposed to alternate 12 h of darkness and light each.

### Preparation of extract

Fresh leaves of the plant material were thoroughly washed with distilled water to remove dirt and soil, and dried under shade and optimal ventilation for 2 weeks. The dried leaves were further chopped into small pieces and reduced to powder using electronic miller and made suitable for maceration extraction procedure with 80 % methanol. The powder was extracted with the same volumes of the solvent in three sequential steps for 3 days each. The mixture then was strained; the marc (the damp solid material) was pressed; after standing decantated and the liquid part was clarified by filtration. Then the extract was evaporated under reduced pressure until all the solvent was removed at 40 °C. Finally percentage yield of hydromethanolic leaves extract of *Indigofera spicata* Forssk. was determined and kept in a refrigerator at 4 °C until use.

### Preliminary phytochemical screening

Standard screening tests of the extract were carried out for various plant chemical constituents; for the presence or absence of secondary metabolites such as terpenes, alkaloids, steroidal compounds, phenolic compounds, tannins, saponins and flavonoids using standard procedures [28, 29].

### Acute toxicity studies

The acute oral toxicity test of the hydroalcoholic leaf extract of *indigofera spicata* Frossk. done according to the limit test of OECD Guideline No.425 [27]. Initially one animal was dosed at 2000 mg/kg. The rat was then kept under strict observation for physical or behavioral changes; especially for the first 4 h continuously and finally overnight mortality was observed. As per the guideline, since there was no any detectable toxicity in the first rat additional 4 female rats were exposed with the test dose and similarly observed for any sign of toxicity. Fourteen days Post exposure toxicity evaluations were done and the change in rats body weight after 3–4 h fasting was determined.

### Evaluation of the effect of the leaves extract of *I. spicata* Forssk. on Blood glucose level

#### Study design

In all models the animal groups and the substance administered were as follows:

- Group I: received 2 % Tween-80 in normal saline (2 % TW80)
- Group II: received 5 mg/kg glibenclamide (5 mg/kg GC)
- Group III: received 100 mg/kg extract (100 mg/kg ISP)
- Group IV: Received 200 mg/kg extract (200 mg/kg ISP)
- Group V: received 400 mg/kg extract (400 mg/kg ISP)

For oral glucose tolerance tests (OGTT), rats were used since they are preferable in such studies [13]. As per the OECD guideline the extract doses to be administered were determined based on the acute toxicity study and volume of administration was 1 ml/100 g of body weight of the animal [27]. As people traditionally use the preparations of the plant extract via oral route using water as a vehicle; the study was conducted using oral route of administration.

In all cases samples for BGL determination were taken by cutting the tip part of the tail of the rodents using aseptic scissors. Glycemia (BGL) was determined by the glucose-oxidase peroxidase method with glucose reagent strips using ACCU-CHEK® ACTIVE electronic glucometer.

A. Effect of *I. spicata* Forssk. extract on BGL of normoglycemic mice
  Healthy normal male mice were fasted for 4–6 h, but water was allowed ad libtum, and then randomly divided into five different groups (6 animals per group). The animals were treated according to their respective grouping as explained above. Using aseptic conditions, blood samples were then collected from tail tips of each animal to determine BGL at 0, 1, 2, 3, 4 and 10 h post treatment.
B. Effect of *I. spicata* Forssk. extract on BGL of oral glucose loaded rats/oral glucose induced by alloxan in the early course of its action tolerance test
  Rats were made fasted overnight for 12–14 h and assigned randomly into 6 groups (*n* = 6), each group in separate cage. Thirty minutes before extract treatment, all of the rats were loaded with 2 g/kg glucose solution orally. Blood samples were collected prior to treatment (i.e. 0 time), 30, 60 and 120 min after administration of extract in order to evaluate their blood glucose level.
C. Effect of *I.spicata* Forssk. extract on BGL of alloxan induced Diabetic mice Experimental induction of diabetes
  Male Swiss albino mice were fasted overnight (12–14 h) and their weight and fasting blood glucose level was recorded. Mice were then made diabetic by a single intraperitoneal injection of alloxan monohydrate (150 mg/kg body weight). Alloxan was given for each animal according to their body weight & freshly dissolved in normal saline just prior to injection. Food and water were allowed to the animals 1 h after alloxan administration. It should also be emphasized that the range of the diabetogenic dose of alloxan is quite narrow and even light overdosing may be generally toxic causing the loss of many animals [11, 12]. This loss is most likely due to kidney tubular cell necrotic toxicity, in particular when too high doses of alloxan are administered.

After 6 h of alloxanization mice were kept for the next 24 h on 5 % glucose solution bottles in their cages to prevent hypoglycemia induced by alloxan in the early course of its action [11].

Three days (72 h) after alloxanization, plasma blood glucose level of each animal was determined and animals with a fasting blood glucose level above 200 mg/dl [30, 31] were included in the study.

Diabetic rodents were kept overnight, each group in a separate cage and were fasted for 4–6 h. The animals were then randomly divided into six groups (*n* = 6) and treated according to their respective group as explained before. Blood samples were collected from the tails of the animals to determine BGL at 0, 2, 4, 6, 8 h post-treatment.

### Bioethical clearance

The experiment was performed according to the animal care and welfare guidelines [32]. The experiment protocols were requested to and approved by School of pharmacy, University of Gondar.

### Statistical analysis

All the values were expressed as mean ± SEM (standard error of means) for six rats per group with percentage calculations of changes of some figures. Statistical analyses were carried out by using SPSS statistical soft ware (version-21). Statistical significance of differences within and between groups was assessed by One-way ANOVA followed by post-hoc Tukey’s Multiple Comparison Test. The value of probability less than 5 % (*P* < 0.05) and 1 % (*p* < 0.001) were considered statistically significant and very significant respectively.

## Result

### Plant extract material

After being dried, the hydromethanolic extract of the leaves of *Indigofera spicata* Forssk. was found greenish brown in color and extensively sticky semisolid at room temperature in its texture. The percentage yield of the dry matter was found 16.72 %.

### Preliminary phytochemical screening

Phytochemical screening was done using colour forming and precipitating chemical reagents on the dried leaves extract of *Indigofera spicata Forssk.* to generate preliminary data on the constituents of the plant extracts. The results obtained from the tests were summarized in Table 1. The chemical tests revealed the presence or absence of major secondary metabolites such as alkaloids, steroidal compounds, phenolic compounds, saponnins and others (Table 1).

**Table 1:** Phytochemical screening test of *Indigofera spicata Forssk*. crude hydroalcoholic leaves extract

Phytochemical	Test	Test result
Alkaloids	Wagner’s test	+
Glycoside	Modified Borntrager’s test	+
Tannins	Ferric chloride test	+
Saponins	Foam test	+
Phytosterols	Salkowski’s test	+
Flavonoids	Lead acetate test	+
Phenols	Ferric chloride test	-
Diterpenes	Copper acetate test	+

+ refers presence and – refers absence

### Oral acute toxicity test

This simplified oral acute toxicity test was done mainly to determine the appropriate safe dose range that could be used for subsequent experiments instead of clarifying all the toxicity profile of the crude extract. Acute toxicity studies conducted revealed that the administration of 2000 mg/kg dose of *Indigofera spicata* Forssk. didn’t produce observable changes in behaviors such as alertness, motor activity, breathing, restlessness, diarrhea, convulsions, coma and disappearance of the animals. Accordingly this extract is safe for up to a dose of [medium lethal dose (LD50) could be greater than] 2 g/kg body weight in rats. The change in their average body weight was 2.2 % gain.

### Evaluation of effects of the extract on BGL of normoglycemic mice

The all over change (i.e. lowering) of BGL was significant (*p* < 0.05) across all time points except at 0 h and 10 h of post exposure. Within group analysis revealed that 2 % TW80 in normal saline treated animals showed significant reduction in BGL at the 3^rd^ hr and 4^th^ hr time points compared to the initial or baseline level. In the 200 mg/kg and 400 mg/kg treated groups intragroup comparison revealed significant (*p* < 0.05) BGL reduction at 3^rd^, 4^th^ and 10^th^ hours of treatment compared to the base line BGL. In this comparison the maximal BGL reduction was 31.4 % (*p* < 0.001) and 27.7 % (*p* < 0.05) at the 10^th^ hr for 200 mg/kg and 400 mg/kg ISP doses respectively. Similar comparison in those treated with 100 mg/kg, none of the BGLs determined at different time points were statistically significant compared to the baseline(0 h) BGL but at 4^th^ hr time point BGL significantly(*p* < 0.05) reduced compared to the 1^st^ hr BGL. The maximum reduction of BGL for those treated with 100 mg/kg ISP extract compared to the baseline was 21.5 % at the 4^th^ hr. Likewise, 5 mg/kg GC brought about very significant (*p* < 0.001) reduction across all time points compared to the initial with a maximum of 36.5 % at 4^th^ hr.

None of the extract treated groups showed significant BGL change compared to the negative control at any time point. Glibenclamide treated group showed significant (*p* < 0.05) BGL reduction at 1^st^ and 2^nd^ hr of post-treatment compared to the 2 % TW-80 treated group. Similarly 5 mg/kg GC treated groups showed significant(*p* < 0.05) BGL reduction both at 3^rd^ and 4^th^ hr compared to 100 mg/kg ISP treated group and at 3^rd^(*p* < 0.05) and 4^th^hr (*p* < 0.001) compared with 400 mg/kg ISP treated group. There was no statistically evident difference in BGL when the different doses of the extract treated groups were compared with each other at all-time points (Table 2).

**Table 2:** Effect of crude hydroalcoholic leaves extract of *I. spicata* on BGL of normoglycemic mice

Group	Blood glucose level(mg/dl)					
	0 h	1 h	2 h	3 h	4 h	10 h
2 % TW-80	101.00 ± 6.03	97.17 ± 4.94	89.50 ± 2.81	84.33 ± 3.89	82.17 ± 2.96	83.17 ± 5.47
5 mg/kg GC	104.00 ± 4.60	74.50 ± 3.46^*©,*^ **	70.17 ± 3.56^*©,*^ **	68.17 ± 2.82**	66.02 ± .62**	72.83 ± 4.24**
100 mg/kg ISP	111.67 ± 7.57	113.17 ± 7.46	104.17 ± 6.10	90.00 ± 4.28	87.67 ± 2.75	89.83 ± 5.38
200 mg/kg ISP	105.50 ± 5.95	92.50 ± 4.78	87.67 ± 4.01	83.17 ± 4.38*	79.50 ± 4.65*	72.33 ± 4.55**
400 mg/kg ISP	118.50 ± 7.53	99.50 ± 5.38	99.67 ± 4.70	87.83 ± 5.26*	91.00 ± 4.30*	85.67 ± 3.52*

Values are mean ± S.E, *n* = 6. ISP = Indigofera spicata. GC = glibeneclamide. TW-80 = tween-80. * = Intra-group comparison with fasting blood glucose level (t = 0 h) with *p* < 0.05 and * * for p < 0.001. © = comparison with the negative controls for p < 0.05 and ©© for p < 0.001

### Evaluation of effect of *the* extract on BGL of oral glucose loaded rats/ (OGTT)

BGL of all groups prior to the administration of the vehicle, glibenclamide and extract (t = 0 min) showed no apparent difference compared to each other. All groups, however, in response to the oral glucose load showed average increment of BGL by 21.56 % at 30 min (1 hour after oral glucose loading). Hyperglycemia with glucose challenge was not significantly brought down with 2 % TW80 at any time point compared to the baseline. None of the extract doses treated groups showed significant difference in BGL amongst each other and compared to the negative control at all time points. The percentage reduction of BGL for 100 mg/kg, 200 mg/kg and 400 mg/kg doses of extract at 120 min compared with the base-line were 9.10, 12.77 and 22.35 % respectively. The intra-group comparison of mean of BGLs at different time points among rats taking 100 mg/kg and 200 mg/kg ISP was insignificant. Whereas 400 mg/kg dose of the extract brought down the BGL of rats at 120^th^ minute of post exposure significantly compared to the initial time (*p* < 0.001), 30^th^ (*p* < 0.001) and 60th (*P* < 0.05) minute BGL of post treatment.

Compared to the negative control group of rats treated with glibenclamide showed statistically significant (*p* < 0.05) reduction of BGL at 60^th^ and 120^th^ minute of post-treatment period. Likewise compared to the base-line and 30^th^ minute changes in BGLs within rates exposed to 5 mg/kg glibenclamide at the 60^th^ and 120^th^ minute of post exposure were statistically significant (*p* < 0.05 and *p* < 0.001 respectively). BGL reduction induced by glibenclamide was statistically significant (*P* < 0.05) at 120^th^ minute of post-exposure with only 100 mg/kg ISP when it was compared with extract treated groups. The summary of effect of the crude extract of *Indigofera spicata Forssk.* leaves on oral glucose tolerance are shown in the next table (Table 3).

**Table 3:** Effect of crude hydroalcoholic leaves extract of *I. spicata* on BGL of rats loaded with oral glucose

Group	Blood glucose level(mg/dl)			
	0	30 min	60 min	120 min
2 % TW-80	119.67 ± 5.43	127.17 ± 5.24	123.17 ± 3.32	106.83 ± 5.19
5 mg/kg GC	123.00 ± 5.18	125.83 ± 9.13	85.83 ± 6.26©*	76.50 ± 5.45©**
100 mg/kg ISP	113.50 ± 6.02	129.17 ± 7.99	111.83 ± 8.39	103.17 ± 8.65
200 mg/kg ISP	116.17 ± 5.52	124.50 ± 7.24	109.17 ± 9.11	101.33 ± 6.31
400 mg/kg ISP	124.50 ± 2.91	127.17 ± 3.97	113.17 ± 4.45	96.67 ± 4.29**

Values are mean ± S.E, *n* = 6. ISP = Indigofera spicata. GC = glibeneclamide. TW-80 = tween-80. * = Intra-group comparison with fasting blood glucose level (t = 0 h) with *p* < 0.05 and _**_ *for p < 0.001. © = comparison with the negative controls for p < 0.05 and ©© for p < 0.001*

### Evaluation of effect of the extract on fasting BGL of diabetic mice

Fifty mice were injected with alloxan and 32 of them were selected as being diabetic in this study, with a success rate of 64.00 %. All the mice selected for this model survived until the end of the experiment. Within groups statistical analysis demonstrated that 2 % TW80 had no significant effect on BGL at all-time points compared to the initial level. In contrast treatment with the extract reduced BGL by less than 25 % (*p* > 0.05) with all dose ranges at 2^nd^ hour of post-treatment followed by significant(*p* < 0.05) reduction(with a maximum of 51.94 % for 400 mg/kg at the 8^th^ hr) across the remaining time points compared to the baseline level. Not only compared to the pretreatment level unlike glybenclamide, BGL reduction of 400 mg/kg ISP extract dose at 6^th^ and 8^th^ hr was also statistically significant(*p* < 0.05) compared to the 2^nd^ hour of post-treatment BGL.

Similarly, except at 2^nd^ hr of post-treatment all the extract doses reduced BGL of mice very significantly (*p* < 0.001) compared to the negative control across all time points. Glibenclamide treated group of mice showed significant (*p* < 0.05 for 2^nd^ hr and *p* < 0.001 for the other time points of post-treatment) BGL changes across all time points compared to both the initial level and the negative control. No statistically significant changes in BGL were observed either amongst the extract or when extract treated groups were compared with the positive control. The mean blood glucose level of fasted animals at various time intervals after p.o. administration of *I.spicata Forssk.* extract on alloxan-induced diabetetic rodents are shown in Table 4.

**Table 4:** Antidiabetic effects of hydroalcoholic leaves extract of *I. spicata* on blood glucose levels in alloxan induced diabetic mice

Group	Blood glucose level(mg/dl)				
	0 h	2 h	4 h	6 h	8 h
2 % TW-80	345.33 ± 29.73	359.67 ± 34.40	405.67 ± 32.48	379.67 ± 21.36	376.00 ± 22.65
5 mg/kg GC	356.50 ± 25.58	246.33 ± 24.15^*©,*^ *	186.50 ± 19.27^*©©,*^ **	169.50 ± 13.19^*©©,*^ **	166.00 ± 10.43^*©©,*^ **
100 mg/kg ISP	356.17 ± 22.12	296.00 ± 23.33	229.67 ± 29.04^*©©,*^ *	223.17 ± 27.91^*©©,*^ *	196.33 ± 20.74^*©©,*^ **
200 mg/kg ISP	331.67 ± 28.39	256.50 ± 28.44	217.50 ± 24.54^*©©,*^ *	200.33 ± 18.78^*©©,*^ *	180.00 ± 14.43^*©©,*^ **
400 mg/kg ISP	373.17 ± 26.97	284.33 ± 22.49*	237.33 ± 20.33^*©©,*^ **	181.50 ± 7.85^*©©,*^ **	179.33 ± 9.95^*©©,*^ **

Values are mean ± S.E, *n* = 6. ISP = Indigofera spicata. GC = glibeneclamide. TW-80 = tween-80. * = Intra-group comparison with fasting blood glucose level (t = 0 h) with *p* < 0.05 and ** for p < 0.001. © = comparison with the negative controls for p < 0.05 and ©© for p < 0.001

## Discussion

Due to complexity and a need of repeated sample taking procedure instead of plasma, capillary whole blood was taken to determine glycemia in this study. Additionally the precision of measuring blood glucose using glucometer is not high, but it is simple and conventionally accepted technique [33, 34]. The success rate of making mice diabetic with alloxan was 64.00 % which might be possibly due to extremely short t1/2 of alloxan in aqueous media [12, 11].

In this study as it is clearly stated the LD50 is greater than 2000 mg/kg body weight of the animal which is in a strong agreement with the LD50 report of leaves extract of *I.pluchra* (LD50 = 2154 mg/kg) even though the leaves and seeds of genus *Indigofera* has reported toxicity [14, 17, 35]. This reflects the wide safety margin of the plant leaves extract as a result doses above the doses used in this study can be used for the evaluation of the effects of the extract on blood glucose levels and possibly for other biochemical parameters [14, 35].

Plants with hypoglycemic and antihyperglycemic activities may contain one or more chemical constituents*.* Preliminary phytochemical screening of the hydromethanolic extract of *Indigofera spicata* as shown in Table 1 contains alkaloids, tannins, flavonoids, glycosides, terpenoids, and others which is largely supported in literature [23].

In line with this finding, in the genus indigofera studies conducted on the leaves of *I. tinctoria, I. arrecta* and *I.pluchra* revealed the presence of alkaloids, flavonoids, saponins, steroids and tannins. And these natural products are known to produce hypoglycemic effects by various mechanisms [36–38]. Especially, Flavonoid and tannins isolated in antidiabetic medicinal plants has been found to stimulate secretion of or possess an insulin like-effect [14–16, 35].

Thus, the significant antidiabetic effect of crude extract of the leaves of *Indigofera spicata* Forssk. could be due to the possible presence of the aforementioned constituents. Some of the bioactive constituents in this study could act synergistically or independently enhancing the activity of glycolytic and glyconeogenic enzymes.

In all of the three models the 2 % TW-80 exposed groups didn’t show significant reduction of BGL compared to the pretreatment level unlike the positive controls and most the extract treated groups. This is a sufficient indicator of changes induced on blood glucose level were attributed to treatments received.

As it is shown in Table 2 the hypoglycemic effect of different doses of the ISP root extract on normoglycemic mice was insignificant compared to the negative controls even though there was decreasement of BGL(by 21.49–24.64 % at 4^th^ hr of post-esxposure to the extract doses) compared to the baseline. Not only the extract treated groups, 2 % TW-80 received group showed reduction of BGL by 18.64 % at similar time point of post-exposure compared to the initial level. But those normoglycemic mice exposed to glibenclamide showed significant reduction of BGL at certain points of time compared to the positive control (*p* < 0.05) and across all time points compared to the baseline (*p* < 0.001). All these reveals that the ISP root extract is devoid of apparent hypogolycemic effect on normoglycemic rodents. This might be due to the inability of the extract to induce hypoglycemia in rodents with normal and intact pancreas which may be further due to the super-dominancy of normal pancreatic function in adjusting the secretion of insulin by which it maintains normal glucose level via counter regulatory physiological mechanisms [39]. And this may largely resemble the anti-hyperglycemic activity of metformin [40].

In case of OGTT, the rise in BGL (average 21.56 %) at 30^th^ minute of substance exposure confirms physiologic induction of hyperglycemia due to oral glucose loading. In this test, glucose tolerance was improved by 20.13 % in 100 mg/kg, 18.61 % in 200 mg/kg and 23.98 % (*p* < 0.001) in 400 mg/kg extract treated groups at 120^th^ minute of post treatment compared to 30^th^ minute level. However, rats treated with the extract doses never showed significant difference in BGL compared to the negative control and even compared to the initial level (except 400 mg/kg dosed groups, *p* < 0.001 at 120 min of post treatment). This might be due to the delayed onset of action, as it is seen in diabetic cases the statistically significant anti-hyperglycemic action of the extract is after 2 h of administration.

As shown on Table 4 and the next figure all doses of the extract significantly reduced BGL of diabetic mice compared to the pretreatment level and the negative control nearly like glibenclamide (except at 2 h). Typically the anti-hyperlycemic activity of 400 mg/kg extract dose was 36.40, 51.36 and 51.94 % at 4^th^, 6^th^ and 8^th^ hr of post treatment respectively compared to the initial value. Similar comparison for glybenclamide treated group shown 47.69, 52.45 and 53.44 % respectively which is almost similar to 400 mg/kg ISP treated group. This enhanced activity unlike on the normoglycemic and oral glucose loaded rodents might be due to the physiologic enhancement/potentiation of the pharmacologic effect of the extract on impaired glucose level or abnormal blood glucose control. From other studies it was reported that the more pronounced effect of the extract in alloxan-induced diabetic mice may possibly be due to the limited or compromised action of insulin in diabetic condition, and conversely a greater and more direct role of the hypoglycemic principle present in the extract [40].

**Fig. 1:**
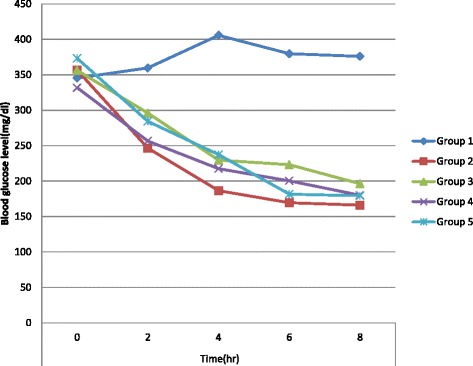
Antidiabetic activity of root extract of *I.spicata Forssk.* on alloxan induced diabetic mice

As it is displayed on Table 4 and according to most of the other data observed the onset of action of the extract is relatively delayed compared to glibeneclamide with a preponderance of effect beginning on 4^th^ hr of post exposure. In addition, the blood glucose lowering activity is most importantly dose dependent. Considering both the normoglycemic and diabetic cases the BGL lowering effect of the crude extract of the plant is delayed in its’ onset and long lasting in its’ duration. In light of this, the antihyperglycemic active principle(s) present within this plant material might be promising to have sustainable blood glucose controlling effect by minimizing risk of fluctuations (Fig. 1).

## Conclusion

The study revealed that the crude hydroalccoholic extract of *ISP* leaves has prominent anti-diabetic effect and can be therefore used as a good insight for new anti-diabetic drug source with a call for further studies. In addition, possible subacute and chronic toxicities needs to be further elaborated within the standard pharmacological protocols, keeping in mind that this plant is locally used by humans for the management of various health problems even if it has reported hepatotoxic and teratogenic effects in animals.
